# An Isoxazoloquinone Derivative Inhibits Tumor Growth by Targeting STAT3 and Triggering Its Ubiquitin-Dependent Degradation

**DOI:** 10.3390/cancers15092424

**Published:** 2023-04-23

**Authors:** Yuanzhu Xie, Shuaiwen Zhu, Ling Chen, Hongdou Liu, Ting Peng, Zhengnan Ming, Zizheng Zou, Xiyuan Hu, Wensong Luo, Kunjian Peng, Yuan Nie, Tiao Luo, Dayou Ma, Suyou Liu, Zhiyong Luo

**Affiliations:** 1 Department of Biochemistry and Molecular Biology, Hunan Province Key Laboratory of Basic and Applied Hematology, Hunan Key Laboratory of Animal Models for Human Diseases, School of Life Sciences, Xiangya School of Medicine, Central South University, Changsha 410008, China; 192501026@csu.edu.cn (Y.X.); 212501022@csu.edu.cn (L.C.); hongdou93691@163.com (H.L.); zouzizheng@csu.edu.cn (Z.Z.); 202501026@csu.edu.cn (X.H.); pkj163@163.com (K.P.);; 2 Xiangya School of Pharmaceutical Sciences, Central South University, Changsha 410013, China; zsw11001@163.com (S.Z.); madayou@csu.edu.cn (D.M.); 3 Hunan Key Laboratory of Oral Health Research, Xiangya Stomatological Hospital, Xiangya School of Stomatology, Central South University, Changsha 410008, China

**Keywords:** quinone, TNBC, STAT3, BCSCs

## Abstract

**Simple Summary:**

We designed and synthesized a series of novel naphthoquinone derivatives, of which ZSW has a high activity to inhibit the growth of tumor cells and low toxicity. We determined that ZSW suppresses triple-negative breast cancer cell activity by targeting STAT3 and provides a new compound structure candidate for TNBC clinical drug development.

**Abstract:**

Background: Triple-negative breast cancer (TNBC) is the most aggressive breast cancer subtype, with shorter five-year survival than other breast cancer subtypes, and lacks targeted and hormonal treatment strategies. The signal transducer and activator of transcription 3 (STAT3) signaling is up-regulated in various tumors, including TNBC, and plays a vital role in regulating the expression of multiple proliferation- and apoptosis-related genes. Results: By combining the unique structures of the natural compounds STA-21 and Aulosirazole with antitumor activities, we synthesized a class of novel isoxazoloquinone derivatives and showed that one of these compounds, ZSW, binds to the SH2 domain of STAT3, leading to decreased STAT3 expression and activation in TNBC cells. Furthermore, ZSW promotes STAT3 ubiquitination, inhibits the proliferation of TNBC cells in vitro, and attenuates tumor growth with manageable toxicities in vivo. ZSW also decreases the mammosphere formation of breast cancer stem cells (BCSCs) by inhibiting STAT3. Conclusions: We conclude that the novel isoxazoloquinone ZSW may be developed as a cancer therapeutic because it targets STAT3, thereby inhibiting the stemness of cancer cells.

## 1. Introduction

Triple-negative breast cancer (TNBC) is the most aggressive subtype of breast cancer because of the poor expression of estrogen receptor (ER), progesterone receptor (PR), human epidermal growth factor 2 (HER2), and high ki-67 status [1]; this means there are a lack of targeted drug therapies compared with the other three subtypes: luminal A, luminal B, and HER2-type [2]. Nowadays, surgical removal, chemotherapy with taxanes, anthracyclins or cisplatin, and radiotherapy are still the main strategies of TNBC treatment, as there is still no clinical target drug therapy for TNBC. Furthermore, most patients relapse after receiving first-line chemotherapy due to chemoresistance [3]. While TNBC only accounts for about 15–20% of all breast cancer cases [4], it has more robust migratory and invasive capabilities to metastasize, resulting in a lower five-year survival rate and a disproportionally higher rate of mortality [5]. Breast cancer stem cells (BCSCs) are a unique class of breast cancer cell properties with self-renewal and differentiation. Several proteins, such as aldehyde dehydrogenase isoform 1(ALDH1), sox2, nanog, and β-catenin, are considered BCSC markers [6]. Targeting BCSCs is thought to be effective in breast cancer treatment, which assists with therapy resistance, metastasis, and tumor recurrence [7]. Therefore, BCSCs’ inhibition offers an opportunity to cure breast cancer.

The signal transducer and activator of transcription 3 (STAT3) plays a vital role in cancer progression [8,9]. Persistent STAT3 activation is observed in many cancer tissues. As for breast cancer, high STAT3 activity is associated with a more aggressive character, while TNBC is associated with uncontrollable STAT3 activation [10]. In fact, STAT3 is activated in over 40% of breast cancer patients [11,12]. STAT3 undergoes phosphorylation at tyrosine 705 (Tyr 705) by interacting with active kinases, especially janus kinase 2 (JAK2), and active STAT3 combines with other STAT3 monomers to form a homo-dimer via the src homology 2 (SH2) domain, and then translocates into the nucleus, promoting gene transcription as a transcription factor [12,13,14]. STAT3 induces the expression of genes such as bcl-2, c-myc, survivin, and cyclin D, which are critical for the proliferation, anti-apoptosis, and chemoresistance of TNBC [15]. STAT3 also acts as an immunotherapy target in many cancers by inhibiting profound immune-stimulating factors, including chemokines (CCL5, CXCL10) and interferons (IFNs) [16,17,18,19].

Quinones are widely found in nature and have broad-spectrum biological activities, such as antibacterial, anti-multiple sclerosis, antimalarial, and anti-inflammatory properties [20,21,22]. Some natural quinone medicines are widely used in clinical cancer chemotherapy for their unique structures, such as daunorubicin [23], doxorubicin [24], STA-21 [25], and 17-AAG [26]. The naturally occurring naphthoquinone Aulosirazole, first isolated from the blue-green alga Aulosira fertilissima by K. Stratmann, indicated tumor-selective cytotoxicity [27]. It was reported that Aulosirazole was a suitable substrate for NQO1 and an effective IDO inhibitor, but its specific mechanism was still unclear, and the synthetic route was very complicated [28]. We designed and synthesized a series of novel quinolineisoxazoles with more straightforward synthesis methods by substituting the isoxazole for the isothiazole of Aulosirazole according to the bioisosterism, expecting them to synthesize anti-tumor drugs with better activity, and selected one of them to explore novel mechanisms.

In this study, we synthesized a series of isoxazoloquinone derivatives and focused on ZSW, which inhibited TNBC stem cells by regulating STAT3 ubiquitination degradation.

## 2. Materials and Methods

### 2.1. General Procedure for the Synthesis of ***2a–2c*** and ***10***

To a solution of substituted aldehydes (5.00 mmol) in methanol (15 mL), hydroxylamine hydrochloride aqueous solution (7.5 mmol) and sodium carbonate (3.75 mmol) were added. The reaction mixture was stirred at 25°C for 1 h under an air atmosphere. After the reaction, the mixture was diluted with water and then extracted with dichloromethane three times. The combined organic layer was dried over anhydrous sodium sulfate, filtered, and evaporated under reduced pressure. The compounds **2a–2c** and **10** were obtained and used directly in the next step without further purification.

### 2.2. 3-Ethylnaphtho[2,3-d]isoxazole-4,9-dione ***4a***

The propionaldehyde (58 mg, 1 mmol) was added to dichloromethane (6 mL) and H_2_O (5 mL). Then, NH_2_OH·HCl (138 mg, 2 mmol) and Na_2_CO_3_ (82 mg, 1 mmol) were added. After stirring for 7.5 h at room temperature, the reaction phases were separated, and the organic phase was the next reaction phase. The naphthoquinone (158 mg, 1 mmol), triethylamine (10 mg, 0.1 mmol), and 5.5% aqueous sodium hypochlorite (2 mL, 1.5 mmol) were added. After stirring for 36 h at room temperature, 100 mL H_2_O was added to the reaction phase and washed with dichloromethane (30 mL × 3). The organic phase was dried with Na_2_SO_4,_ and the solvent was removed under reduced pressure. The residue was purified with a silica gel column and was eluted with PE/EA = 80:1 to obtain **4a** (105 mg, 46.24%).

^1^H NMR (500 MHz, CDCl_3_) *δ* 8.30–8.28 (m, 1H), 8.26–8.25 (m, 1H), 7.89–7.83 (m, 2H), 3.13 (q, *J* = 7.5 Hz, 2H), 1.44 (t, *J* = 7.5 Hz, 3H). ^13^C NMR (126 MHz, CDCl_3_) *δ* 179.54, 173.35, 165.26, 163.46, 135.08, 134.30, 133.41, 132.30, 127.49, 127.27, 120.35, 19.38, 11.82. HRMS (ESI) *m*/*z* [M+H]^+^ calcd. for C_13_H_10_NO_3_^+^, 228.0655; found 228.0655

### 2.3. 3-(3,4,5-Trimethoxyphenyl)-naphtho[2,3-d]isoxazole-4,9-dione ***4b***

Naphthoquinone (63 mg, 0.4 mmol), triethylamine (4 mg, 0.04 mmol), and 5.5% aqueous sodium hypochlorite (0.545 mL, 0.4 mmol) were added to dichloromethane (5 mL), and then the 3,4,5-trimethoxybenzaldehyde oxime (84 mg, 0.4 mmol) was added. After stirring for 24 h at room temperature, 100 mL H_2_O was added to the reaction phases and washed with dichloromethane. The organic phase was dried with Na_2_SO_4_, and the solvent was removed under reduced pressure. The residue was purified with a silica gel column and eluted with petroleum PE/EA = 8:1 to obtain **4b** (112 mg, 76.98%).

^1^H NMR (500 MHz, CDCl_3_) *δ* 8.28 (ddd, *J* = 7.5, 6.0, 1.5 Hz, 2H), 7.90–7.83 (m, 2H), 7.63 (s, 2H), 4.00 (s, 6H), 3.96 (s, 3H). ^13^C NMR (126 MHz, CDCl_3_) *δ* 178.71, 173.31, 166.48, 160.67, 153.25, 140.64, 135.31, 134.32, 133.85, 131.60, 127.91, 127.18, 121.27, 119.49, 106.77, 77.30, 77.04, 76.79, 60.95, 56.34. HRMS (ESI) *m*/*z* [M+H]^+^ calcd. for C_20_H_16_NO_6_^+^, 366.0972; found 366.0980.

### 2.4. 3-(3,4,5-Trimethoxybenzyl)naphtho[2,3-d]isoxazole-4,9-dione ***4c***

Naphthoquinone (84 mg, 0.53 mmol), triethylamine (5 mg,0.053 mmol), and 5.5% aqueous sodium hypochlorite (1 mL, 0.74 mmol) were added to dichloromethane (7 mL), and then the 3,4,5,-trimethoxyphenylacetaldehyde oxime (120 mg, 0.53 mmol) was added. After stirring for 3d at room temperature, the reaction phases were separated, and the aqueous phase was extracted with dichloromethane. The organic phase was dried with Na_2_SO_4_ and the solvent was removed under reduced pressure. The residue was purified with a silica gel column and was eluted with petroleum PE/DCM = 1:4 to obtain **4c** (160 mg, 79.41%).

^1^H NMR (500 MHz, CDCl_3_) *δ* 8.27 (d, *J* = 7.3 Hz, 1H), 8.24 (d, *J* = 7.3 Hz, 1H), 7.88–7.82 (m, 2H), 6.74 (s, 2H), 4.37 (s, 2H), 3.88 (s, 6H), 3.82 (s, 3H). ^13^C NMR (126 MHz, CDCl_3_) δ 179.27, 173.16, 165.37, 161.02, 153.29, 137.07, 135.13, 134.38, 133.37, 132.26, 131.02, 127.51, 127.27, 120.14, 106.34, 60.80, 56.15, 31.40. HRMS (ESI) *m*/*z* [M+H]^+^ calcd. for C_21_H_18_NO_6_^+^, 380.1129; found 380.1137.

### 2.5. 5,7-Dibromo-8-quinolinol ***6***

To a solution of 8-hydroxyquinoline (5.8 g, 40.00 mmol) and sodium bicarbonate (3.36 g, 40.00 mmol) in methanol (70 mL), diluent of bromine (16 g, 100 mmol) in methanol was slowly added drop-by-drop. The reaction mixture was stirred at 25°C for 5 min under an air atmosphere. After the reaction, 10% sodium thiosulfate solution was added to quench the unreacted bromine. The mixture was filtered, resulting in compound **6** (12 g, 99%).

### 2.6. 7-Bromoquinoline-5,8-dione ***7***

To a solution of 5,7-dibromo-8-quinolinol **6** (2.33 g, 7.70 mmol) in concentrated sulfuric acid (20 mL), concentrated nitric acid (1 mL, 15.4 mmol) diluted with concentrated sulfuric acid was slowly added drop-by-drop at 0 °C for 30 min. After the reaction, the mixture was diluted with ice water, and then extracted with dichloromethane three times. The combined organic layer was dried over anhydrous sodium sulfate, filtered, and evaporated under reduced pressure. The residue was purified by column chromatography with a mixed eluent of petroleum ether and ethyl acetate (5:1, *v*/*v*), resulting in compound **7** (1.456 g, 79.5%).

### 2.7. 3-(3,4,5-Trimethoxyphenyl)isoxazolo [4,5-g]quinoline-4,9-dione ZSW

To a solution of 7-bromoquinoline-5,8-dione **5** (70.00 mg, 0.30 mmol) in dichloromethane (8 mL), triethylamine (3 mg, 0.03 mmol) and 5.5% aqueous sodium hypochlorite (0.4 mL, 0.3 mmol) were added at 25°C for 36 h. After the reaction, the mixture was diluted with ice water, and then extracted with dichloromethane three times. The combined organic layer was dried over anhydrous sodium sulfate, filtered, and evaporated under reduced pressure. The residue was purified by column chromatography with a mixed eluent of petroleum ether and ethyl acetate (3:1, *v*/*v*), resulting in the target compound ZSW (24 mg, 21.96%). Compound **11** was also obtained by the same synthetic method.

^1^H NMR (500 MHz, CDCl_3_) δ 9.17 (dd, J = 4.5, 1.5 Hz, 1H), 8.67 (dd, J = 8.0, 1.5 Hz, 1H), 7.84 (dd, J = 8.0, 4.5 Hz, 1H), 7.64 (s, 2H), 4.03 (s, 6H), 3.98 (s, 3H). ^13^C NMR (126 MHz, CDCl_3_) δ 177.43, 171.38, 166.61, 160.72, 155.05, 153.37, 147.54, 140.95, 135.98, 131.05, 128.49, 120.91, 119.43, 106.81, 61.00, 56.39. HRMS (ESI) m/z [M+H]^+^ calcd. for C_19_H_15_N_2_O_6_^+^, 367.0925; found 367.0930.

### 2.8. 4-(3-Bromopropoxy)-3,5-dimethoxybenzaldehyde ***9***

To a solution of 4-hydroxy-3,5-dimethoxybenzaldehyde (182 mg, 1.00 mmol) and K_2_CO_3_ (276 mg, 2.00 mmol) in acetonitrile (50 mL) was added 1,3-dibromopropane (732 mg, 3.00 mmol). The reaction mixture was stirred at 45°C for 24 h under air atmosphere. After the reaction was completed, removed potassium carbonate by suction filtration, and dried acetonitrile off, the mixture was diluted with water, and then extracted with ethyl acetate three times. The combined organic layer was dried over anhydrous sodium sulfate, filtered and evaporated under reduced pressure.the residue was purified by column chromatography with a mixed eluent of petroleum ether and ethyl acetate (8:1, *v*/*v*) affording compound **9** (240 mg, 80.0%).

### 2.9. Synthesis of Biotin–ZSW

To a solution of **11** (472 mg, 1.00 mmol) and K_2_CO_3_ (138 mg, 1.00 mmol) in DMF (50 mL), D-biotin (243 mg, 1.00 mmol) was added. The reaction mixture was stirred at 25 °C for 36 h under an air atmosphere. After the reaction, the mixture was diluted with water and then extracted with ethyl acetate three times. The combined organic layer was dried over anhydrous sodium sulfate, filtered, and evaporated under reduced pressure. The residue was purified by column chromatography with a mixed eluent of petroleum ether and ethyl acetate (3:1, *v*/*v*), resulting in biotin–ZSW as an orange solid. (330 mg, 52.0%).

^1^H NMR (500 MHz, DMSO) *δ* 9.10 (d, *J* = 3.9 Hz, 1H), 8.58 (d, *J* = 7.7 Hz, 1H), 7.95 (s, 1H), 7.53 (s, 2H), 6.42 (s, 1H), 6.34 (s, 1H), 4.32–4.20 (m, 3H), 4.11 (s, 1H), 4.04 (t, *J* = 5.7 Hz, 2H), 3.89 (s, 6H), 3.07 (s, 1H), 2.80 (dd, *J* = 12.4, 4.9 Hz, 1H), 2.57 (d, *J* = 12.4 Hz, 1H), 2.32 (t, *J* = 7.3 Hz, 2H), 1.97 (m, *J* = 12.1, 6.1 Hz, 2H), 1.57 (m, *J* = 14.8, 7.4 Hz, 3H), 1.46 (m, *J* = 13.8, 5.5 Hz, 1H), 1.34 (m, *J* = 14.6, 6.8 Hz, 2H). ^13^C NMR (126 MHz, DMSO) *δ* 178.43, 173.37, 171.86, 167.78, 163.15, 160.40, 154.69, 153.54, 148.40, 139.45, 135.78, 131.45, 128.93, 121.57, 118.87, 107.08, 69.81, 61.49, 61.37, 59.64, 56.63, 55.81, 33.81, 29.49, 28.47, 25.01. HRMS (ESI) *m*/*z* [M+H]^+^ calcd. for C_31_H_33_N_4_O_9_S^+^, 637.1890; found 637.1965.

### 2.10. Cell Line Culture and Viability Assay

All cell lines were purchased from American Type Culture Collection (ATCC, Manassas, VA, USA). MDA-MB-231 cells were cultured in DMEM/F-12 medium, while BT-549 cells and HCC1937 cells were cultured in RPMI medium with 10% fetal calf serum (FBS) (Biological Industries, Cromwell, CT, USA) and 1% penicillin/streptomycin. 184B5 cells were cultured in RPMI medium with 10% horse serum, hydrocortisone (0.5 μg/mL), human recombinant EGF (10 ng/mL), and bovine insulin (5.0 μg/mL). MDA-MB-231 and HCC1937 cell mammospheres were planted in ultra-low-adherent 6-well plates (BIOFIL cat:001006) with mammosphere medium (MammoCult™ Human Medium Kit for the culture of human mammospheres and tumorspheres, STEMCELL, Vancouver, Canada, cat,#05620) according to the manufacturer’s instructions. Cells were plated in 96-well plates with 3500 cells in each well, adding the drug at 12 h and assaying the cell proliferation 2 days after drug addition by the MTT experiment.

### 2.11. In Vivo Studies

All animal protocols were reviewed and approved by the Department of Laboratory Animals, Central South University. Four-to-six weeks old female BALB/c nude mice were purchased from SJA Laboratory Animal Co., Ltd, Changsha, China. MDA-MB-231 cells were washed two times with PBS, re-suspended in PBS, and injected subcutaneously at 0.1 mL into nude mice. Cisplatin or ZSW was dissolved in DMSO, PEG300, and Tween 80(1:1:1) at a concentration of 5 mg/mL by sonication. When the tumors grew to ~1000 mm^3^, the mice were euthanized, and hearts, livers, spleen, lungs, kidneys, and tumors were harvested, cryopreserved, or immersed in 10% formalin. Body weight and tumor size were measured by an electronic balance or electronic vernier caliper every 2 days before drug injection. All the procedures were evaluated and approved by the Institutional Animal Care and Use Committee of Central South University.

### 2.12. Western Blot Analysis and Antibodies

Whole protein was extracted using RIPA lysis buffer (Beyotime, Beijing, China). The Pierce™ Rapid Gold BCA (Thermo Fisher, Waltham, MA, USA, cat.A53225) was used to measure the protein concentration. Membranes were incubated with diluted antibodies in 5% *w*/*v* BSA or 5% non-fat milk, 1×PBST, 0.1% Tween 20 at 4 °C with gentle shaking overnight. The primary antibodies used were β-Catenin (cat.#8480, Cell Signaling, Danvers, MA, USA, 1:1000), Sox2 (cat.#3579, Cell Signaling, 1:1000), Stat3 (cat.#9139, Cell Signaling, 1:1000), p-Stat3 (Tyr705) (cat.#9145, Cell Signaling, 1:1000), Stat1 (cat.#14994, Cell Signaling, 1:1000), HA-Tag (cat.AE008, ABclonal, Woburn, MA, USA, 1: 1000), DDDDK-Tag (cat.AE005, ABclonal, 1:1000), RCC1 (cat.sc-55559, Santa Cruz Biotechnology, Dallas, TX, USA, 1:500).

### 2.13. Plasmids

293T cells were transfected with pLentiCRISPR v2 plasmids (Sino Biological Inc., Beijing, China) with sgRNAs targeting STAT3, which were purchased as previously described [29]. STAT3 ORF cDNA expression plasmid, C-Flag tag and C-HA tag (Sino Biological Inc.), PMD2G, and PSPAX2 were transfected into 293T cells. The supernatant containing the virus was collected and filtered after 48 h and mixed with fresh medium. Puromycin was used to remove noninfected MDA-MB-231 cells.

### 2.14. Luciferase Assay

293T cells were spread in a 6-well plate, and plasmid pSTAT3-TA-luc (cat.D2259, Beyotime) and pRL-TK (cat.D2760, Beyotime) were co-transfected by using Neofect™ DNA transfection reagent (Neofect Beijing Biotech Co., Ltd., Beijing, China) after 12 h. Firefly luciferase and renilla luciferase were measured using a double-luciferase reporter assay kit (cat.FR201, Transgen, Quezon City, Philippines), following the manual.

### 2.15. Isothermal Titration Calorimetry

The human STAT3 amino acid (127–722) with 6×His tag fragments was cloned into a pET-28a plasmid and then transformed and expressed in E. coli BL21(DE3). A total of 1 mM Isopropyl β-D-Thiogalactoside (IPTG) was added to LB medium and OD_600_ = 0.5 E. coli bacteria were lysed by sonication and centrifuged for 5 min at 12,000× *g*. The recombinant proteins were purified after incubating the supernatant with Ni-NTA (QIAGEN) for 2 h at room temperature. ITC Studies were performed at 25 °C. Briefly, ZSW, previously suspended in DMSO, was diluted in PBS. The final DMSO concentration was 5%, and the STAT3 was 0.37g/L. Three hundred microliters (300 μL) of ~5 μM STAT3 was placed in the cell and titrated with 50 μM inhibitors. Titrations took place by injecting 2.5 μL inhibitor in a 5 min injection after the titration peak returned to the baseline. The KD was calculated using the TA-ITC analysis software (TA-I TECHNOLOGY CO., LTD., Taiwan, China), using the one-site model. The titration of the buffer into STAT3 was a control experiment as background noise from the measurements.

### 2.16. Molecular Docking

A molecular docking study was executed by using AutoDock4.2. The X-ray crystal structure of STAT3 was downloaded from the Protein Data Bank (PDB code: 1BG1), water was removed using Pymol, and conditioning was performed using AutodockTools by adding all polar hydrogen atoms. The three-dimensional structure of ZSW was built, hydrogenated, and energy-minimized using MarvinSketch. The grid box was centered on the STAT3 monomer, and the grid box size was adjusted to include the STAT3 SH2 domain. Other parameters were kept at default. Finally, the pictures were generated using Pymol.

### 2.17. ALDH1 Activity Assay

ALDH1 activity was determined using an ALDEFLUOR™ Kit (STEMCELL cat.#29902).

### 2.18. Statistical Analysis

All data were shown as mean ± SD and analyzed using GraphPad Prism (Version 8; La Jolla, CA, USA). Differences were analyzed using the *t*-test or Tukey–Kramer post hoc test. Differences at *p* < 0.05 were considered to be statistically significant.

## 3. Results

**Figure 1:**
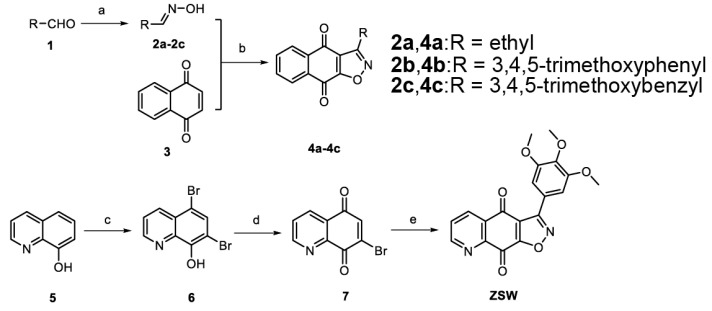
Synthesis of isoxazoloquinone derivatives ZSW.

1. The synthesis of isoxazoloquinone derivative ZSW:

The synthetic route of the compounds is depicted in Figure 1. Firstly, the aldoximes **2a**–**2c** were obtained by the reaction of substituted aldehyde **1** and hydroxylamine hydrochloride in the presence of sodium carbonate, according to the reported method [30]. A new class of isoxazoloquinone derivatives **4a**–**4c** were synthesized from the oxidation and cyclization of 1,4-naphthoquinone **3** with **2a**–**2c**, respectively. Then, 8-hydroxyquinoline **5** was bromized by Br_2_ to obtain dibromo intermediate **6**, with reference to the method of Yu, Qian et al. [31]. 7-bromoquinoline-5,8-dione **7** was subsequently prepared from the oxidation of 5,7-dibromo-8-quinolinol **6**, according to the literature [31]. Finally, the cyclization of **7** with 3,4,5-trimethoxybenzaldoxime **2b** provided the isoxazoloquinone derivative ZSW in good yield.The structure of the target compounds was further determined by NMR and HRMS spectra.

2. Structure–activity relationship (SAR) analysis:

The bioactivities of target compounds **4a**–**c** and ZSW are shown in Table 1. Compared to STA-21 and S3I-201, the antiproliferative activity of most compounds against human TNBC cells significantly increased. 3-aromatic substituent **4b** showed much stronger cell growth inhibitory activity than the corresponding naphthylquinones containing 3- alkyl substituent **4a**. The antiproliferative activity was obviously reduced when the carbon chain between the 3-aromatic substituent and the isoxazole ring was extended as displayed with the IC_50_ of **4c**. When naphthoquinone products **4b** were replaced with quinolinequinone products ZSW, the antiproliferative effect in vitro was markedly enhanced. ZSW also showed a strong inhibitory effect on doxorubicin-resistant MDA-MB-231 cells, which is a first-line clinical chemotherapy small molecule.

**Figure 2:**
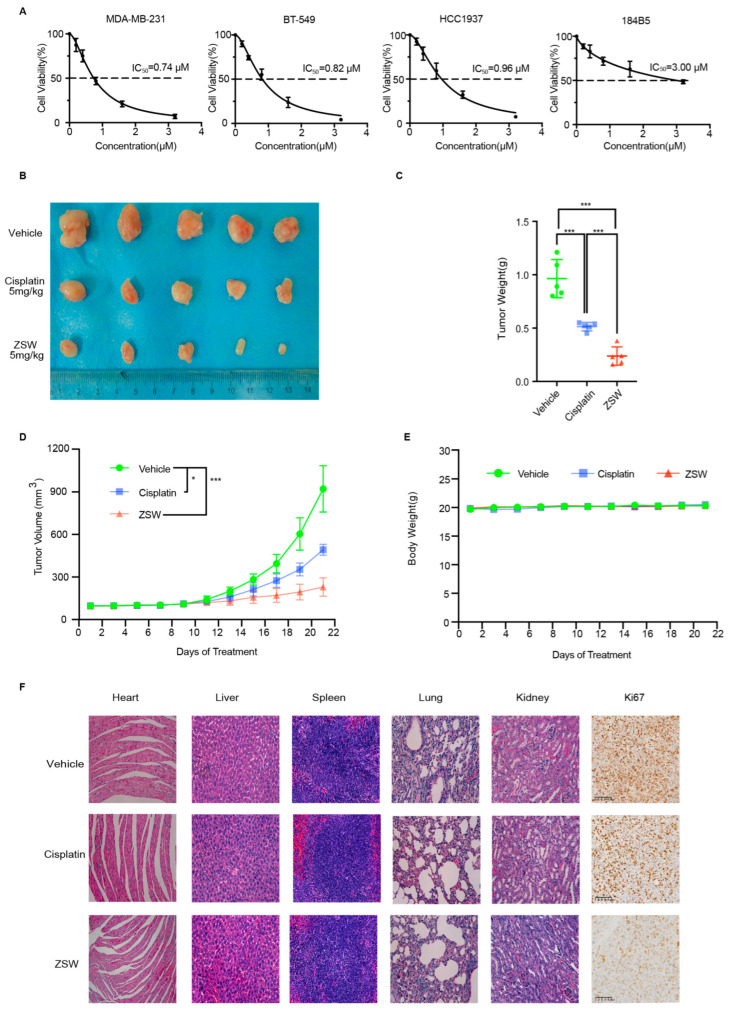
ZSW inhibits the proliferation of TNBC cells. (**A**) ZSW reduces the numbers of MDA-MB-231 and BT-549 cell clone formation but with a lighter inhibition of 184B5 cells. (**B**) The nude mice were treated with a vehicle which included phosphate-buffered saline, polyethylene glycol 300, and dimethyl sulfoxide (1:1:1 by volume), cisplatin (5 mg/kg), or ZSW (5 mg/kg), dependently. Tumors were stripped and weighed. The weights were represented as the mean tumor weight ±  SD (**C**). The tumor volume (**D**) and body weight (**E**) changes were measured. (**F**) Representative images of heart, liver, spleen, lung, kidney, and tumor tissue sections from vehicle-, cisplatin-, and ZSW-treated mice, staining by hematoxylin, eosin, or ki67 antibody (scale bar, 200 μm). * *p* < 0.05, compared with vehicle control; *** *p* < 0.001, compared with vehicle control.

3. ZSW inhibits the proliferation of TNBC cells:

ZSW strongly inhibited TNBC cell lines, such as MDA-MB-231, BT-549, and HCC1937 (Table 1), with an IC_50_ below 1 μM at 48 h. However, it slightly inhibited the normal epithelial cell line 184B5 (Figure 2A and Figure S1), which showed that ZSW had some selective impacts among TNBC cells and normal epithelial cells. A nude mouse tumor xenograft growth model was created to determine the antitumor potential of ZSW in vivo, and cisplatin was chosen as a positive control. ZSW showed promising activity in inhibiting MDA-MB-231 TNBC cells compared with cisplatin in 5 mg/kg in vivo (Figure 2B). ZSW was more effective in suppressing the weight of xenograft tumors compared to cisplatin (Figure 2C). Tumor volume and the weight of mice were recorded at the indicated time points (Figure 2D,E). ZSW exhibited a more significant volume inhibition than cisplatin when treating with the same concentration. In contrast, the weight of mice barely changed between the three groups. The tissue sections of mice, such as heart, liver, spleen, lung, kidney, and tumor tissues, were examined using HE or immunohistochemical staining for morphological analysis. Mice treated with ZSW had no significant histopathologic changes in their tissues, accompanied by a decrease in ki67 expression in tumor tissues (Figure 2F).

Overall, ZSW showed a potent inhibition of the proliferation of TNBC in nude mice with a tolerated concentration. Next, we sought to determine the mechanism of the tumor-suppressive action of ZSW.

**Figure 3:**
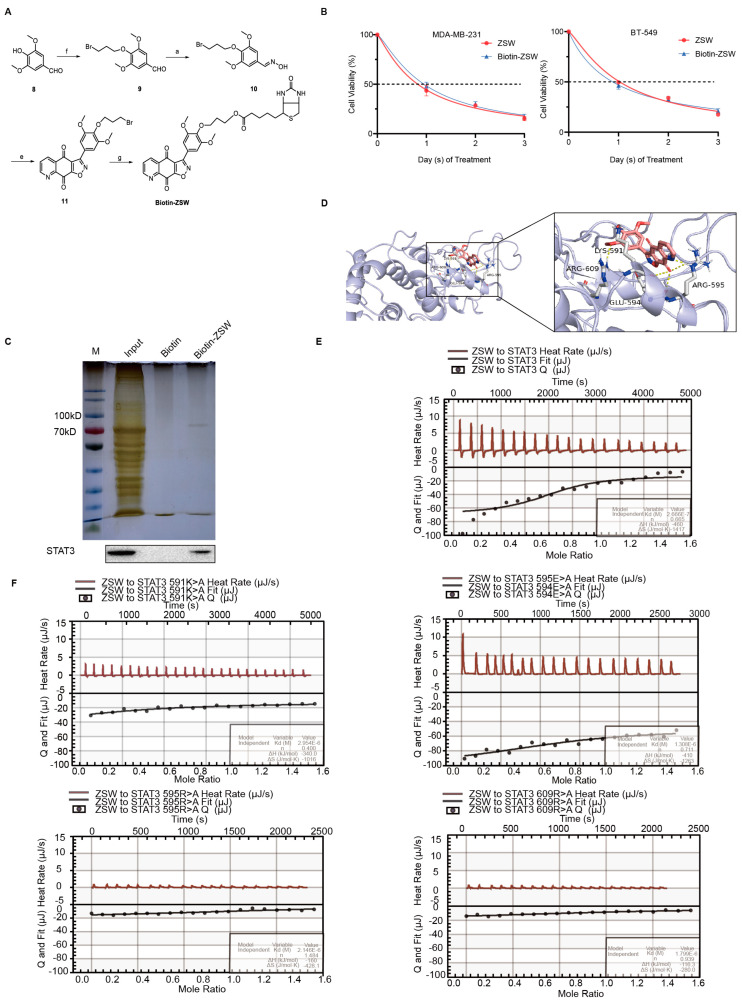
ZSW has a direct binding with the STAT3 SH2 domain. (**A**) Synthesis of biotin–ZSW. Reagents and conditions: a. NH_2_OH·HCl, Na_2_CO_3_, CH_3_OH:H_2_O = 1:1, rt, 1 h; b. NaClO, TEA, DCM, rt; c. Br_2_, NaHCO_3_, CH_3_OH, rt, 5 min, 99.0%; d. HNO_3_, H_2_SO_4_, 0 °C, 0.5 h, 79.5%; e. NaClO, DCM, rt, 36 h, 22.0%; f. 1,3-Dibromopropane, K_2_CO_3_, Acetonitrile, 45 °C, 24 h, 80.0%; g. Biotin, K_2_CO_3_, DMF, rt, 36 h, 52.0%; (**B**) Bio–ZSW has a similar inhibition activity in TNBC cells with ZSW. (**C**) Silver staining of proteins pulled down by biotin–ZSW and ZSW, and Western blotting of pull-down lysis using STAT3 antibody. The uncropped blots are shown in File S1. (**D**) Molecular docking study of ZSW binding to the STAT3 SH2 domain (PDB code 1BG1). The study was generated by AutoDock4.2, and the figures were generated by Pymol. Hydrogen bonds were shown as yellow dashed lines. Amino acid residues of STAT3 involved in the hydrogen bonding interactions are shown in the picture. (**E**) Isothermal titration calorimetry (ITC) measurements of the binding of ZSW to STAT3 (127–722) or ZSW to STAT3 (127–722) mutant (**F**). The line is the fit of the binding isotherm to extract the thermodynamic parameters using a one-site binding model.

4. ZSW has a direct binding with STAT3:

To further verify the target protein induced by ZSW treatment in MDA-MB-231 cells, we linked the ZSW with biotin. We obtained biotin–ZSW, which has a similar cancer-suppressive ability to ZSW (Figure 3A,B), and identified the proteins that bind to biotin–ZSW specifically using LC/MS. We determined that STAT3 was specifically stripped, most likely followed by Western blotting verification (Figure 3C). We performed a computational docking experiment between ZSW and the STAT3 SH2 domain using AutoDock4.2. ZSW occupied the pTyr705 binding site and side pocket, which are related to the disruption of STAT3 phosphorylation and the dimerization of inhibitors, and formed four hydrogen bonds with the residues Lys591, Glu594, Arg 595, and Arg609 (Figure 3D). Next, we constructed a prokaryotic expression plasmid pET28a-STAT3 to prepare recombinant STAT3 protein. STAT3 proteins were successfully expressed in the prokaryotic expression system after IPTG induction, but were present in inclusion bodies. The recombinant STAT3 protein containing N-terminal 6 × His-Tag was purified by nickel column affinity (Figure S2). Isothermal titration calorimetry was used to investigate the interaction between ZSW and STAT3. The dissociation constants were calculated from equilibrium binding, and each binding component is given for the presented ITC curves, which showed a direct binding between ZSW and STAT3. The binding strength reduced when the residues mutated (Figure 3E,F).

**Figure 4:**
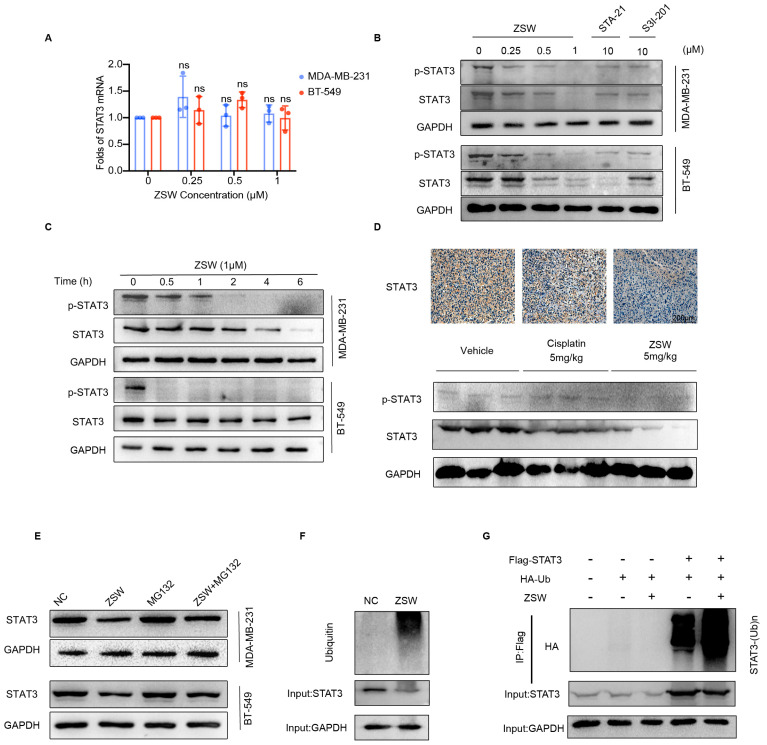
ZSW promotes STAT3 degradation via the ubiquitin–proteasome system. (**A**) ZSW has little impact on decreasing the mRNA level of STAT3. (**B**) MDA-MB-231 and BT-549 cells were treated with DMSO, ZSW, STA-21, or S3I-201 for 48 h (**C**), and in a time-dependent manner both in MDA-MB-231 cells and BT-549 cells. (**D**) ZSW decreases the p-STAT3 and the t-STAT3 levels in vivo via Western blot. Immunohistochemistry staining of MDA-MB-231 cell tumor tissues for the expression of STAT3 (scale bar, 200 μm). (**E**) MDA-MB-231 and BT-549 cells were treated with DMSO or ZSW in the presence and absence of MG132 (10 μM) for 12 h, and cytoplasmic extracts were prepared and analyzed by Western blot using STAT3 antibody. (**F**) The MDA-MB-231 cells were exposed to ZSW for 12 h, and then input or immunoprecipitated cell lysates were incubated with STAT3, GAPDH, or ubiquitin Abs, respectively. (**G**) The MDA-MB-231 cells were co-transfected with pcDNA3.1-STAT3-Flag and pcDNA3.1-Ub-HA plasmids, and were exposed to compound ZSW for 12 h, and then cell lysates were immunoprecipitated with anti-Flag Abs, respectively. ns, *p* > 0.05, compared with DMSO control. The uncropped blots are shown in File S1.

5. ZSW promoted STAT3 degradation through ubiquitination:

Next, we detected the mRNA level of STAT3. ZSW did not repress the STAT3 RNA level, but showed intense repression of phosphor-STAT3 (p-STAT3) and total-STAT3 (t-STAT3) at the same time (Figure 4A). ZSW down-regulated the level of t-STAT3 and p-STAT3 in a dose-dependent way (Figure 4B) and a time-dependent way (Figure 4C) in TNBC cells. The p-STAT3 had a sharp down-regulation after 0.5 h, dealing with 1 μM ZSW, and the t-STAT3 was decreased after about 6 h. We next examined the expression of STAT3 in vehicle mice, confirming that the t-STAT3 and the p-STAT3 level were both reduced by ZSW but barely reduced by cisplatin, using Western blot, and that ZSW attenuated STAT3 expression in the tumor tissue of mice treated with cisplatin or ZSW using a STAT3 antibody, through immunohistochemistry (Figure 4D).

To determine the STAT3 degradation induced by ZSW, we investigated the effect of ZSW in the presence and absence of cycloheximide (CHX) in TNBC cells. The results showed that ZSW promotes CHX-mediated protein synthesis inhibition (Figure S3A). The degradation of STAT3 can be rescued by the proteasome inhibitor MG132 (Figure 4E), but can not be rescued by the lysosome inhibitor Bafilomycin A1 (Figure S3B), which showed that ZSW inhibits the STAT3 level through the ubiquitin proteasome pathway. We then examined STAT3 ubiquitylation and found that it actively ubiquitylated after ZSW treatment by immunoprecipitation (Figure 4F,G).

**Figure 5:**
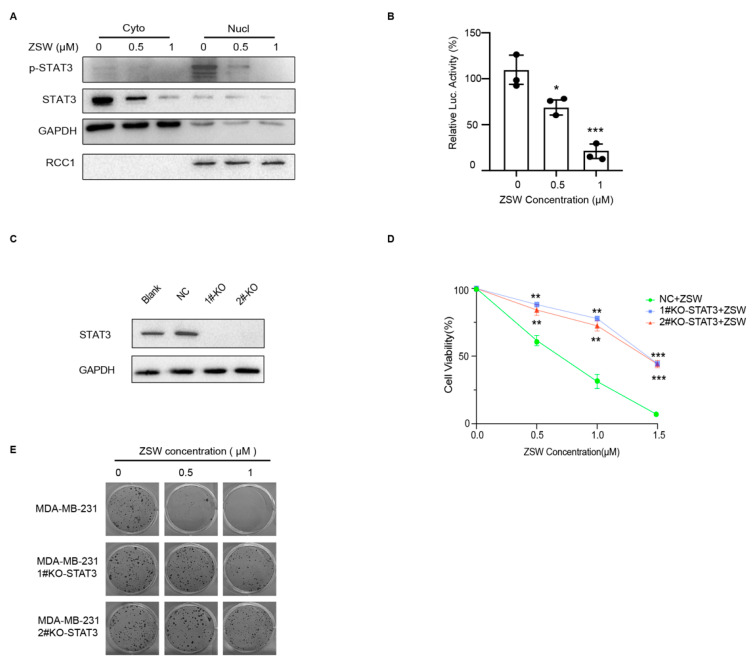
ZSW inhibits TNBC cell proliferation through STAT3. (**A**) ZSW decreases t-STAT3 in the cytoplasm and p-STAT3 in the nucleus. Cyto, cytoplasm; Nucl, nucleus. Glyceraldehyde 3-phosphate dehydrogenase (GAPDH) and regulator of chromosome condensation 1 (RCC1) are used for quantification, respectively. (**B**) ZSW reduced the transcriptional effect of STAT3 by double-luciferase assay. (**C**) MDA-MB-231 cells were transfected with or without lentiCRISPR-STAT3 plasmid, and STAT3 Abs were used to confirm that STAT3 has been completely knocked out. MDA-MB-231 or STAT3 knockout (STAT3^−/−^) MDA-MB-231 cells were incubated with ZSW for 48 h, the viability of which was determined by the MTT assay (**D**) and cloning assay (**E**) as described before. * *p* < 0.05, compared with vehicle control; ** *p* < 0.01, compared with control; *** *p* < 0.001, compared with control. The uncropped blots are shown in File S1.

6. ZSW exerts anti-tumor activity through STAT3:

We extracted cytosolic and nuclear fractions, and Western blots were performed. The p-STAT3 level of MDA-MB-231 cells in the nucleus, along with the t-STAT3 level in the cytoplasm, were both decreased by ZSW (Figure 5A). ZSW significantly reduced the luciferase activity of STAT3, which acts as a transcription factor (Figure 5B). Then, we explored whether lacking STAT3 reduced the sensitivity of MDA-MB-231 to ZSW. STAT3-knockout MDA-MB-231 via CRISPR-Cas9 system cells were harvested as recommended before using a lenti-CRISPR plasmid (Figure 5C), which could decrease the MDA-MB-231 inhibition caused by ZSW (Figure 5D,E).

7. ZSW suppresses the TNBC stem-cell-like properties

**Figure 6:**
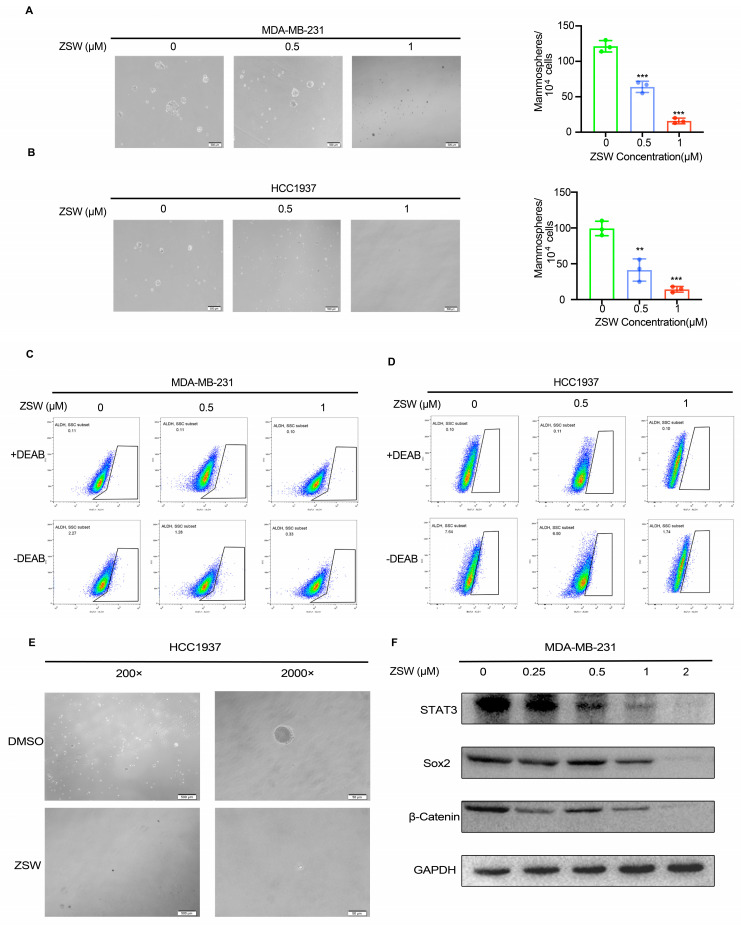
ZSW suppresses breast cancer stem-cell-like properties. Effect of ZSW on MDA-MB-231 and HCC1937 mammosphere formation. MDA-MB-231 (1 × 10^5^ cells/mL) (**A**) and HCC1937(1 × 10^5^ cells/mL) (**B**) were cultured in serum-free suspension conditions in ultralow attachment plates after dealing with the presence or absence of ZSW (0.5 or 1 μM) for 48 h. The number and volume of mammospheres were quantified by optical microscopy (scale bar, 200 μm, ** *p* < 0.01, *** *p* < 0.001). After exposure to ZSW (0.5 or 1 μM, 48 h), the percentage of aldehyde dehydrogenase isoform 1-positive (ALDH1+) section of MDA-MB-231 cells (**C**) and HCC1937 cells (**D**) is shown in the respective panels. ALDH1 activity was evaluated by flow cytometry. (**E**) Changes in cellular morphology in HCC1937 cells after ZSW (1 μM) treatment for 48 h as seen through phase contrast microscopy (scale bar, 500 μm for 200×; 50 μm for 2000×). Some typical markers of TNBC stem cells were measured by Western blotting (**F**). ** *p* < 0.01, compared with control; *** *p* < 0.001, compared with control. The uncropped blots are shown in File S1.

We next sought to determine whether ZSW can suppress stem-cell-like properties in TNBC cells. MDA-MB-231 and HCC1937 cells were plated in 6-well plates and treated with or without ZSW for 48 h. Equal numbers of cells were replated with serum-free suspension culture medium in ultralow attachment plates, and the cell concentrations of the different groups were adjusted to be the same (1 × 10^5^ cells/mL). As shown in Figure 6A,B, the size and number of mammospheres significantly decreased after ZSW treatment. The tumorspheres were shrunk, the edges were not neat, and the cells were fused when a high magnification was provided (Figure 6E). TNBC cells were treated with or without ZSW for 48 h and then digested by collagenase, and ALDH+, and ALDH− populations were measured by flow cytometry. ZSW decreased the ALDH activity of TNBC cells in 48 h (Figure 6C,D). ZSW also attenuated some stem cell markers, such as Sox2 and β-catenin, which are linked with drug resistance (Figure 6F).

## 4. Discussion

Triple-negative breast cancer (TNBC) is a specific molecular subtype that is more malignant due to poor prognosis, wild metastasis, and ease of recurrence. At this stage, surgical excision, radiotherapy, and chemotherapy using cisplatin, taxol, or doxorubicin are the primary treatment for TNBC. However, it has the poorest overall patient survival and the most severe toxic side-effects in therapeutic modality among all breast cancer subtypes because of a lack of targeted therapy agents [32]. Some proteins and microRNAs have been identified as markers of TNBC for abnormal expression or activation, and STAT3 is thought to be one of the most promising targets of TNBC [33].

Constitutive activation and high expression of STAT3 are observed in many cancers; it functions as an oncogene, promoting cancer cell proliferation, migration, and immune escape, and inhibiting apoptosis. Interleukin-6 (IL6), an inflammatory factor, is a major activator of STAT3, which forms an IL-6R/IL-β (gp130) complex and then activates the JAK/STAT3 signaling pathway [34]. JAK activates STAT3 through phosphorylation at Tyr-705 and Ser-727; however, Tyr-705 is necessary for STAT3 activation, and Ser-727 modification reinforces the activity. High levels of active STAT3 form as STAT1/STAT3 heterodimers or STAT3/STAT3 homodimers and translocate into nuclei, regulating the expression of several downstream target genes as transcription factors. The change in STAT3 leads to abnormal cell proliferation and malignant metastasis in breast cancer, and disrupting STAT3 activation progression causes an apparent inhibiting of breast cancer effect [35]. Notably, STAT1 is often considered a tumor suppressor because STAT1-deficient mice have been shown to be more prone to spontaneous and chemically induced tumor formation than wild-type mice, so there is an urgent need to find specific target inhibitors of STAT3 [36].

BCSCs are a small functional characteristic of breast cancer cells, which are linked with a high ability of chemoresistance and radioresistance for the aberrant expression of acetaldehyde dehydrogenase 1 (ALDH1), ATP binding cassette (ABC) transporters, sox2, oct4, and other stemness proteins [37,38]. Among others, the NF-κB pathway, Notch pathway, and Wnt/β-catenin pathway are frequently active in BCSCs [39]. The population of BCSCs has increased after months of chemotherapy and radiotherapy in breast cancer patients, causing a more resistant effect to standard treatments. The proliferation and formation of spheres cultured with a serum-free medium is one of the main phenotypic characteristics of cancer stem-like cells. BCSCs can be enriched in a stem-cell medium as mammospheres cultured by the suspension culture method [40]. Nowadays, several enzyme or protein inhibitors and activators have decreased the rate of BCSCs [41,42]. Small molecular compounds display BCSC inhibitory ability and reduce the proliferation of drug-resistant cells. Furthermore, active STAT3 has recently been suggested as a BCSC promotor [43]. STAT3 inhibitors such as BBI608 display BCSC inhibition and thus have a prominent place in the study of stem cell therapy [44]. ZSW significantly inhibited the formation of mammospheres and the expression of ALDH1, suggesting that ZSW is a potent therapeutic agent for BCSCs.

Quinone derivates represent a large family of compounds and have diverse biological functions. The synthesis route of Aulosirazole was long, and the conditions of some reaction steps were very harsh [28], while our synthetic route of Aulosirazole derivatives was short, and the conditions were mild. We combined the structural characteristics of quinone derivatives STA-21 and Aulosirazole to result in a novel quinolineisoxazole drug through substitution, oxidation, and cyclization reactions, thereby obtaining ZSW. However, we also noted that the inhibition of ZSW to TNBC proliferation did not completely fail after the knockdown of STAT3 by CRISPR-Cas9, which may be due to the fact that the quinone compound ZSW has more complex mechanisms, such as through the IDO1 pathway, in addition to enhancing the STAT3 ubiquitination degradation pathway [45].

Over the past few years, some small molecules have been identified, such as Stattic [46], BP-1-102 [47] and S3I-201 [48], and proteolysis-targeting chimera (PROTAC) technology has demonstrated a new inhibitor strategy [49]. For example, the STAT3 nucleotide inhibitor AZD9150 has demonstrated some oncogenic effects in lung and lymphoma patients, and BBI608, a STAT3 inhibitor, can inhibit the proliferation of cervical cancer primary cells in patients [50,51]. Still, as far as we know, a high level of conspecificity and selectivity of clinical STAT3 inhibitors still appears to be missing.

In short, we have shown a novel quinone derivative ZSW to be a STAT3 inhibitor, binding with the SH2 domain of STAT3, causing STAT3′s ubiquitination and reducing the p-STAT3 level and the t-STAT3 level, thereby inhibiting the proliferation of TNBCs both in vivo and in vitro. ITC data show that ZSW is a high-affinity STAT3 inhibitor, which means that a lower concentration of ZSW displays greater inhibitory efficacy. Moreover, ZSW decreases the number and size of the mammospheres, reduces the expression of some BCSC markers, including ALDH1, β-catenin, and Sox2, in TNBCs, and shows better inhibition of doxorubicin-resistant MDA-MB-231 cells, which suggests that ZSW inhibits growth in chemoresistant TNBC cell lines.

## 5. Conclusions

An isoxazoloquinone derivative, ZSW, inhibits TNBC growth and alleviates BCSCs by targeting STAT3.

## Figures and Tables

**Table 1:** Anti-proliferation on TNBC cell lines of target compounds.

Compound	MDA-MB-231 (IC_50_^a^ in µM)	MDA-MB-231-Doxorubicin Resistance (IC_50_^a^ in µM)	BT-549 (IC_50_^a^ in µM)	HCC1937 (IC_50_^a^ in µM)
**4a**	5.14 ± 0.43	4.45 ± 0.73	4.35 ± 0.95	4.27 ± 0.65
**4b**	2.38 ± 0.23	2.56 ± 0.75	1.95 ± 0.45	1.96 ± 0.24
**4c**	>10	>10	>10	>10
ZSW	0.74 ± 0.06	0.48 ± 0.07	0.82 ± 0.06	0.96 ± 0.09
STA-21	>10	>10	>10	>10
S3I-201 Doxorubicin	>10 0.38 ± 0.04	>10 1.18 ± 0.24	>10 0.37 ± 0.03	>10 0.49 ± 0.09

^a^ Data shown are the means of three independent experiments.

## Data Availability

All data generated or analyzed during this study are included in this published article.

## Supplementary Materials

The following are available online at https://www.mdpi.com/article/10.3390/cancers15092424/s1, Figure S1: ZSW reduces the numbers of MDA-MB-231 and BT-549 cell clone formation but with a slighter inhibition of 184B5 cells, Figure S2: The prokaryotic expression and purification of wild type STAT3 and mutant STAT3 protein., Figure S3:CHX or BAF cannot rescue the down-regulation of STAT3 caused by ZSW. File S1: Full pictures of the Western blots.
