# Supplementary figures and images for: An Isoxazoloquinone Derivative Inhibits Tumor Growth by Targeting STAT3 and Triggering Its Ubiquitin-Dependent Degradation
Source: Cancers (Basel). 2023 Apr 23;15(9):2424. doi: 10.3390/cancers15092424 (PMC10177496; doi:10.3390/cancers15092424)

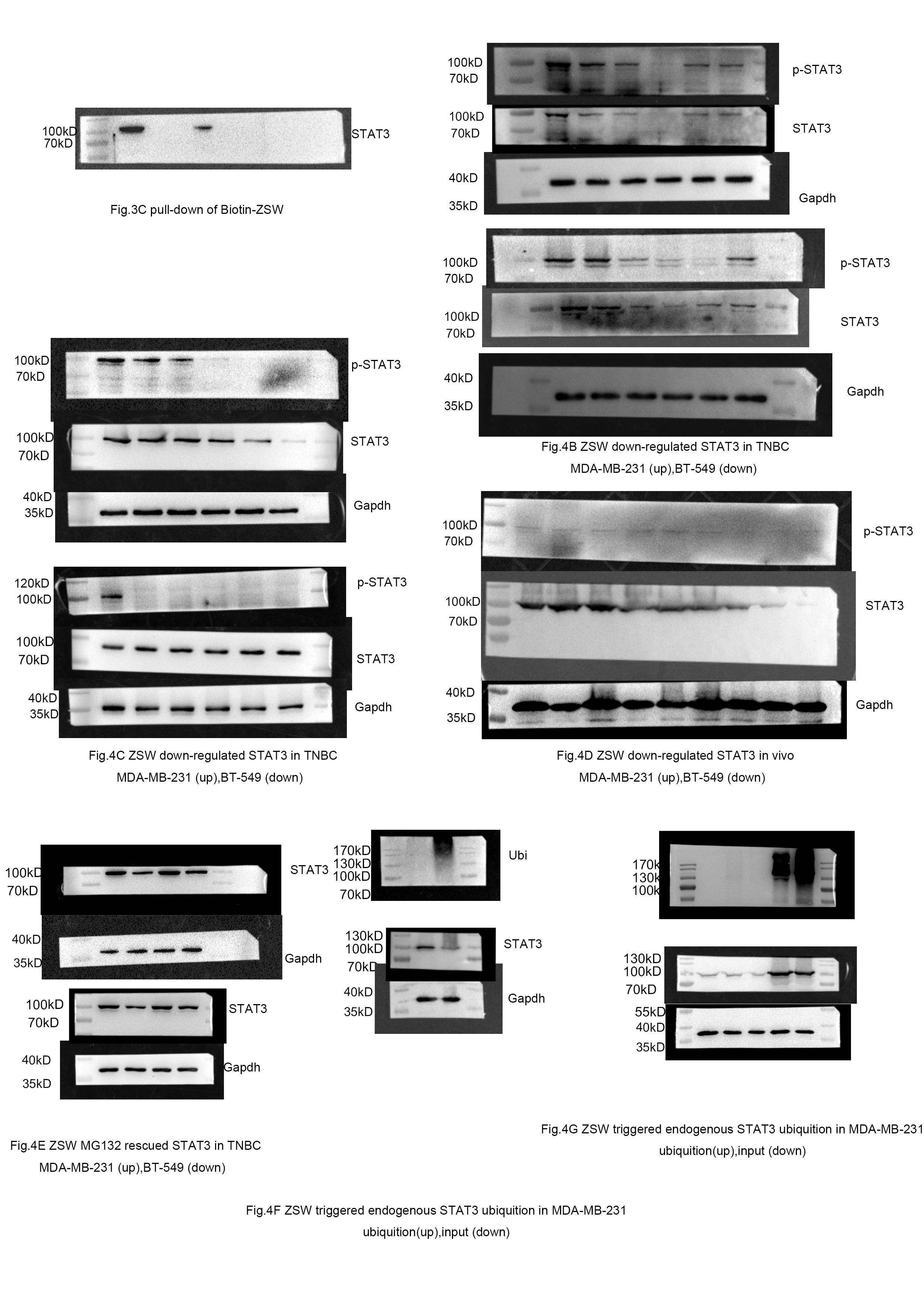

Supplement: Supplementary file 1 [file cancers-15-02424-s001.zip › File S1. uncropped/un-cropped Figures S3-S4 Wb_.png]

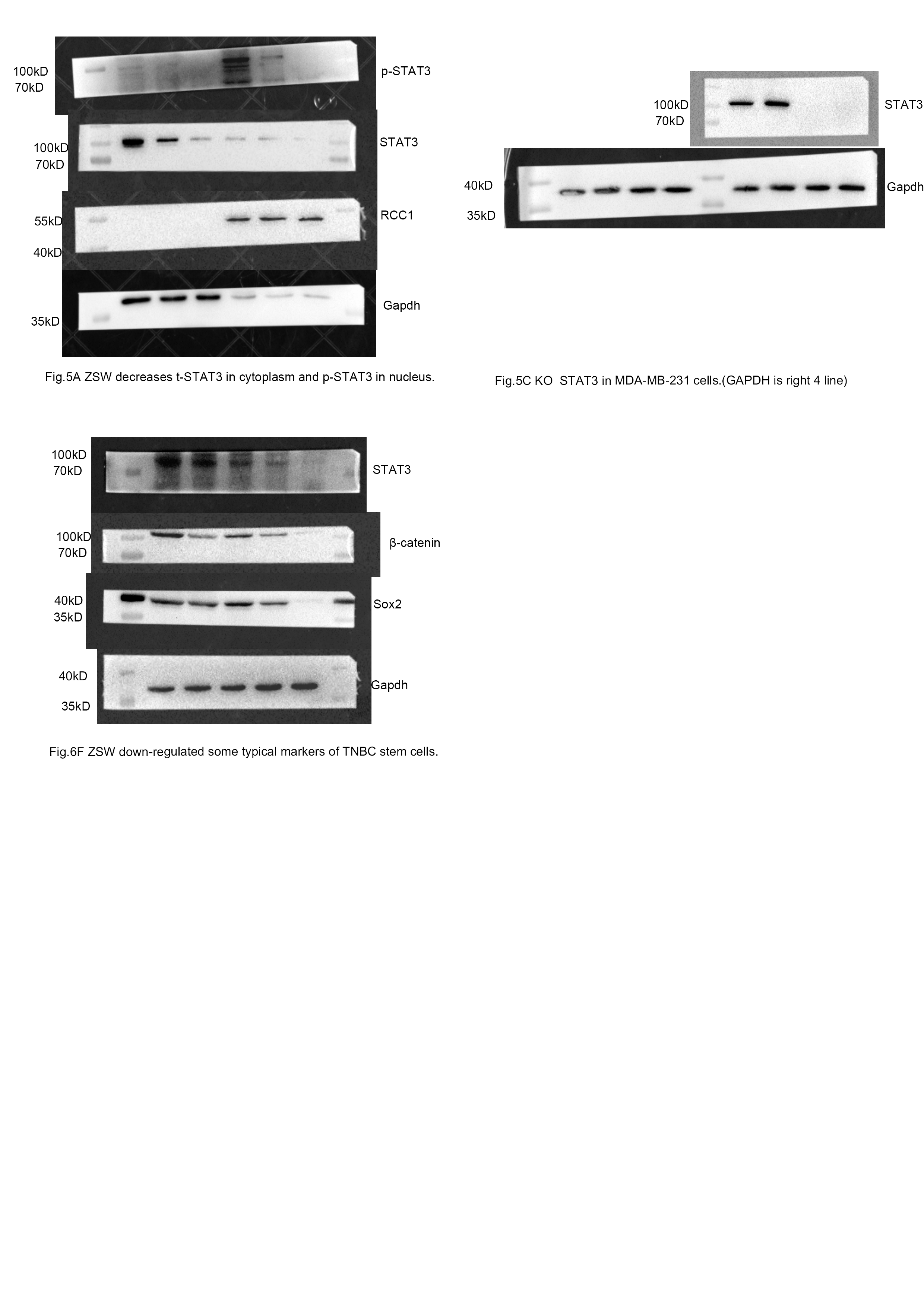

Supplement: Supplementary file 1 [file cancers-15-02424-s001.zip › File S1. uncropped/Uncropped Figures S5-S6 WB.png]

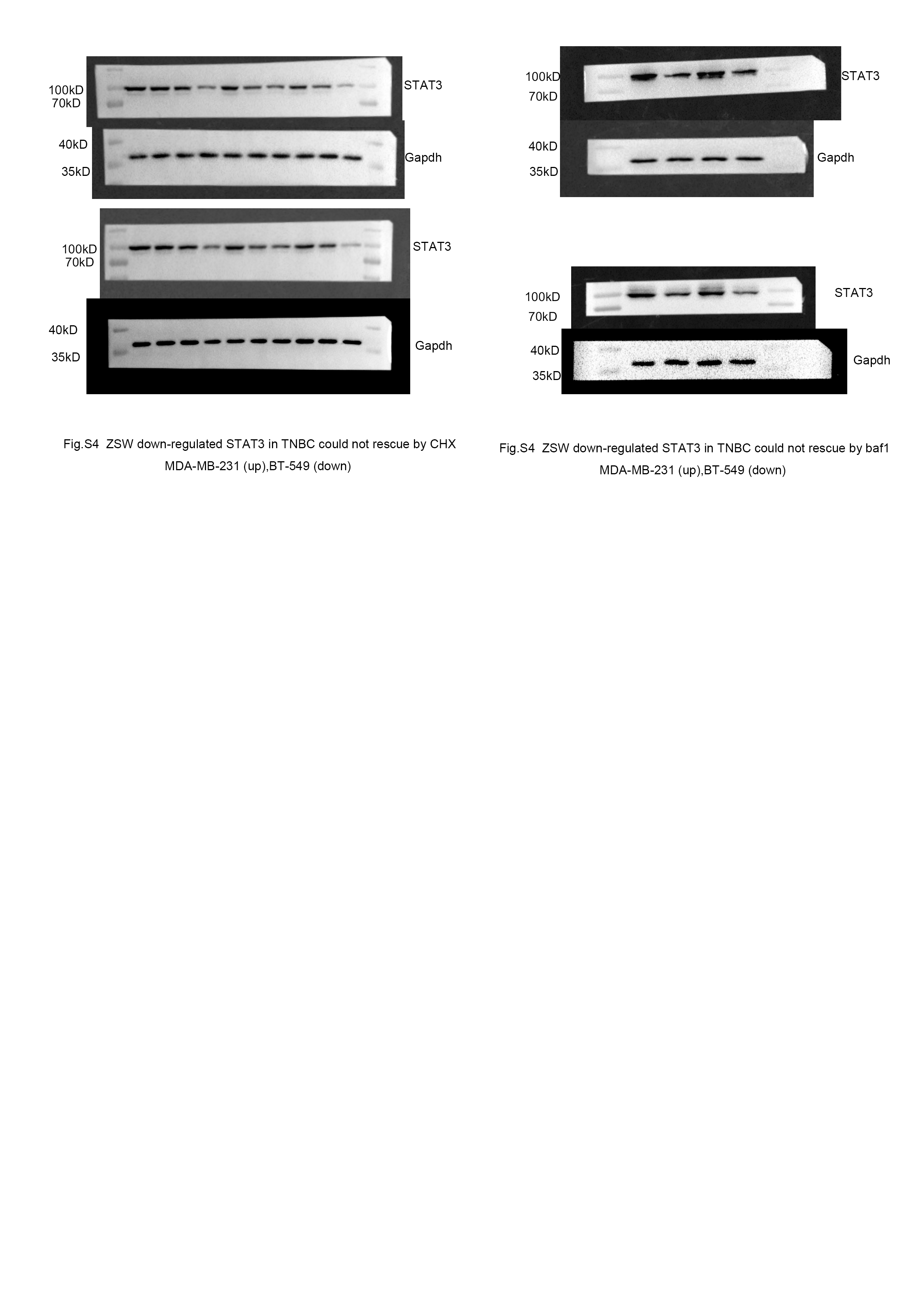

Supplement: Supplementary file 1 [file cancers-15-02424-s001.zip › File S1. uncropped/uncrppoed Figure S4.png]

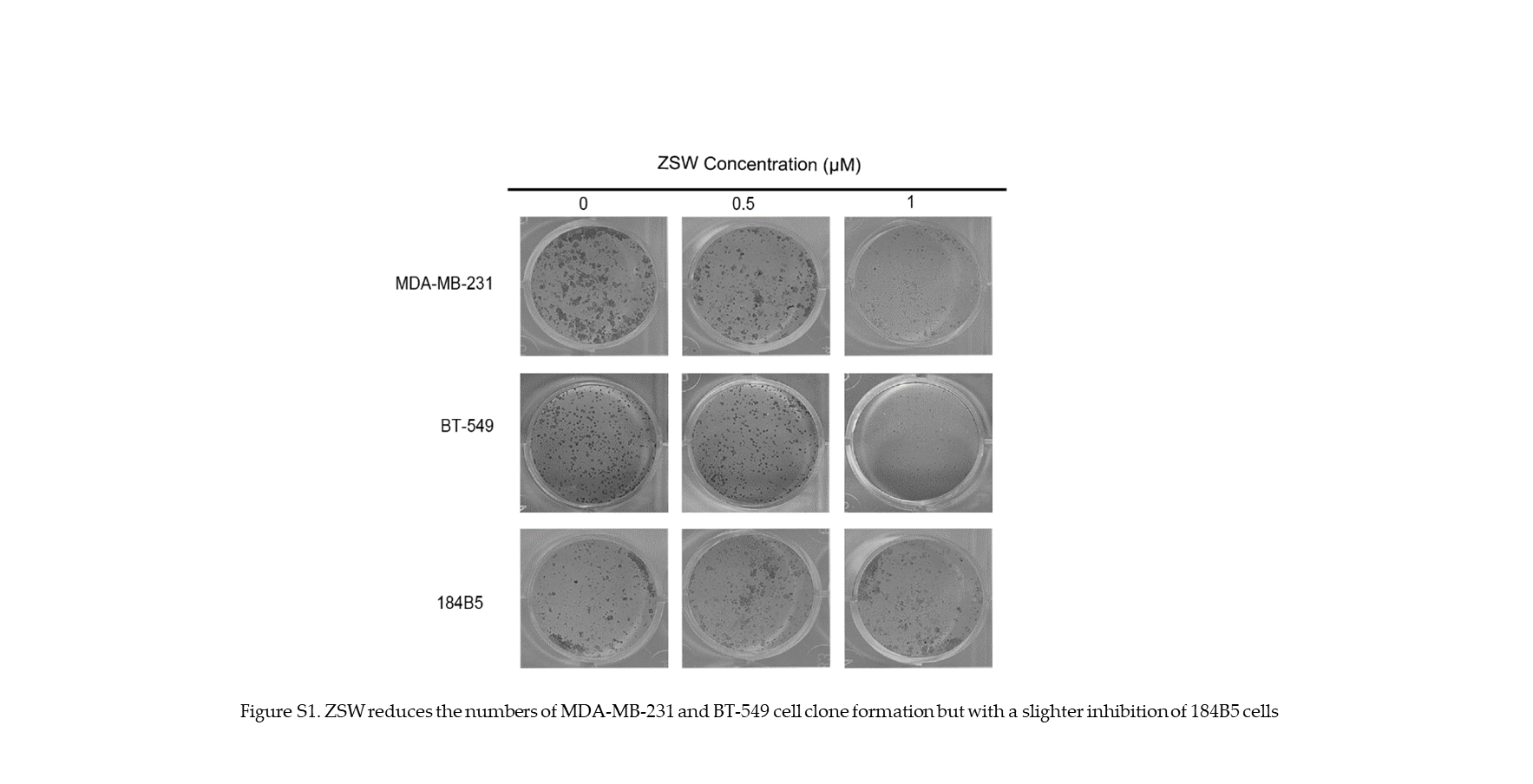

Supplement: Supplementary file 1 [file cancers-15-02424-s001.zip › Supplementary Figure S1.png]

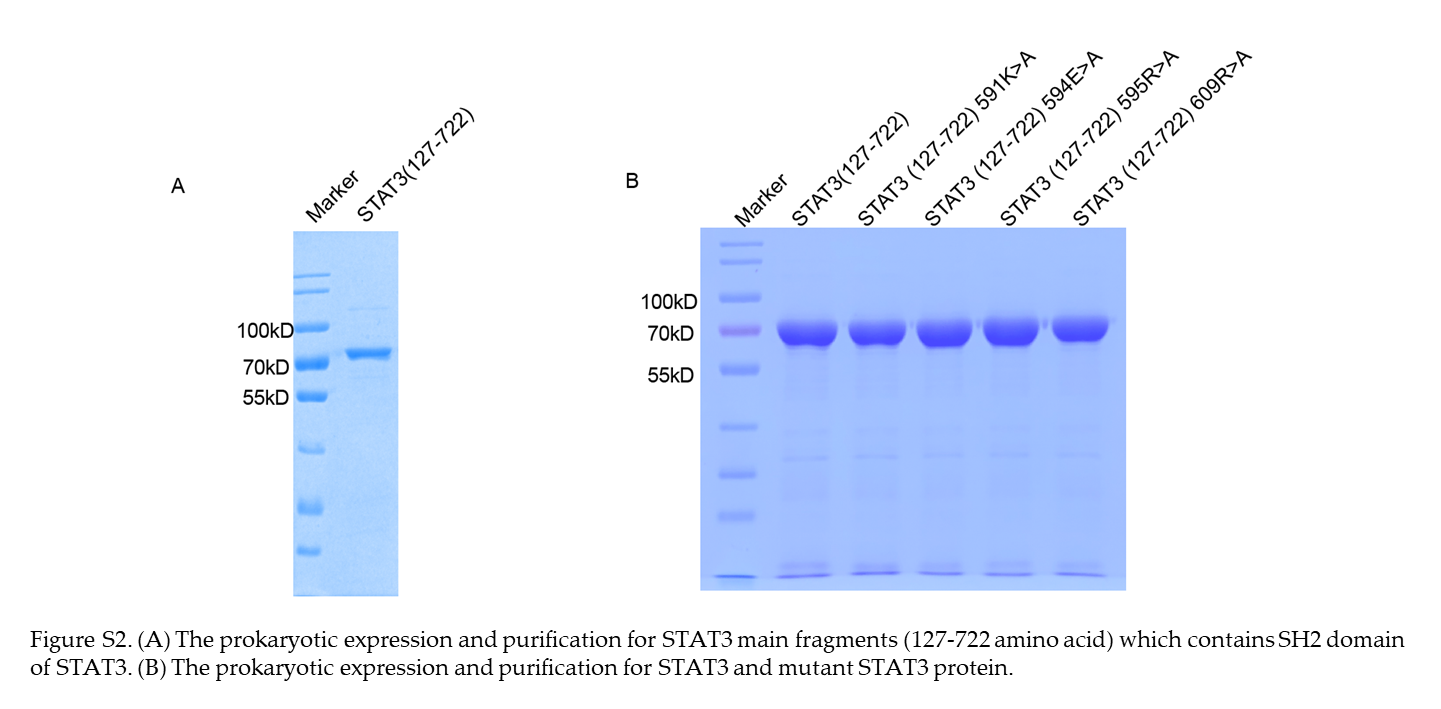

Supplement: Supplementary file 1 [file cancers-15-02424-s001.zip › Supplementary Figure S2.png]

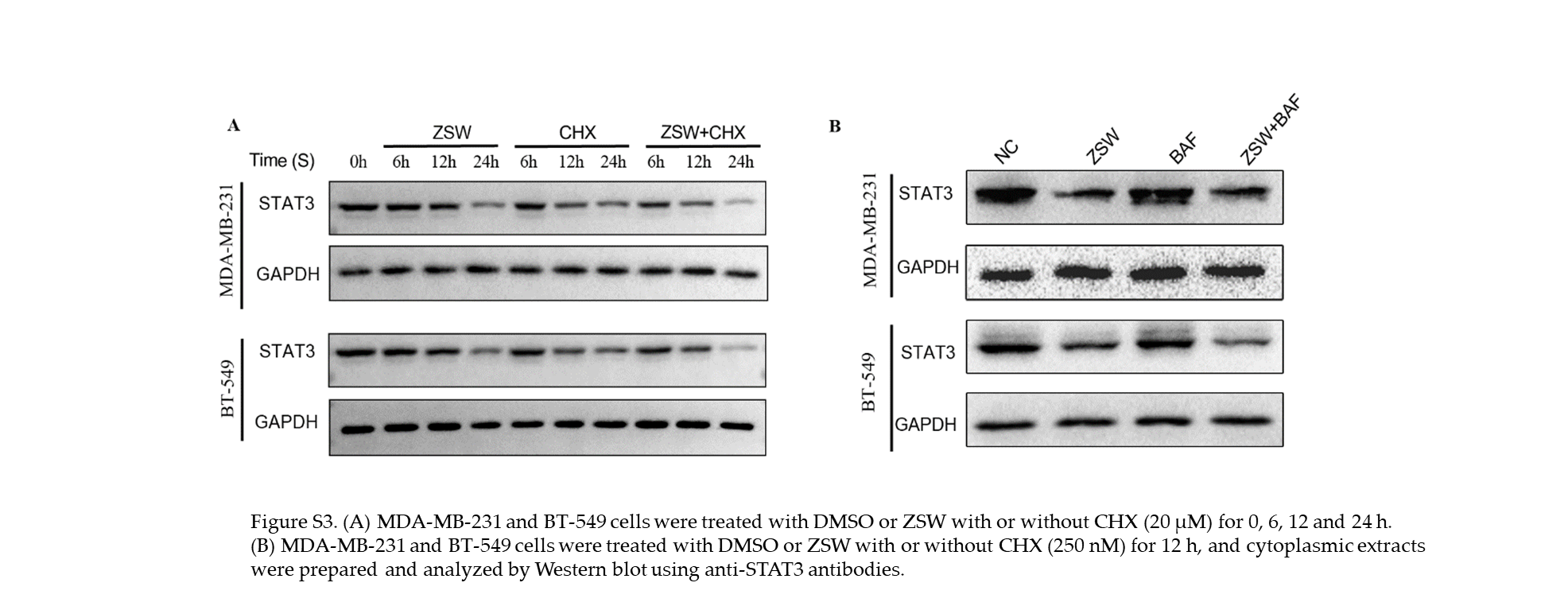

Supplement: Supplementary file 1 [file cancers-15-02424-s001.zip › Supplementary Figure S3.png]
